# Epigenetic memory contributing to the pathogenesis of AKI-to-CKD transition

**DOI:** 10.3389/fmolb.2022.1003227

**Published:** 2022-09-21

**Authors:** Fumiaki Tanemoto, Masaomi Nangaku, Imari Mimura

**Affiliations:** Division of Nephrology and Endocrinology, The University of Tokyo Graduate School of Medicine, Tokyo, Japan

**Keywords:** epigenetic memory, hypoxic memory, AKI-to-CKD transition, RNA polymerase II, transcriptional memory

## Abstract

Epigenetic memory, which refers to the ability of cells to retain and transmit epigenetic marks to their daughter cells, maintains unique gene expression patterns. Establishing programmed epigenetic memory at each stage of development is required for cell differentiation. Moreover, accumulating evidence shows that epigenetic memory acquired in response to environmental stimuli may be associated with diverse diseases. In the field of kidney diseases, the “memory” of acute kidney injury (AKI) leads to progression to chronic kidney disease (CKD); epidemiological studies show that patients who recover from AKI are at high risk of developing CKD. The underlying pathological processes include nephron loss, maladaptive epithelial repair, inflammation, and endothelial injury with vascular rarefaction. Further, epigenetic alterations may contribute as well to the pathophysiology of this AKI-to-CKD transition. Epigenetic changes induced by AKI, which can be recorded in cells, exert long-term effects as epigenetic memory. Considering the latest findings on the molecular basis of epigenetic memory and the pathophysiology of AKI-to-CKD transition, we propose here that epigenetic memory contributing to AKI-to-CKD transition can be classified according to the presence or absence of persistent changes in the associated regulation of gene expression, which we designate “driving” memory and “priming” memory, respectively. “Driving” memory, which persistently alters the regulation of gene expression, may contribute to disease progression by activating fibrogenic genes or inhibiting renoprotective genes. This process may be involved in generating the proinflammatory and profibrotic phenotypes of maladaptively repaired tubular cells after kidney injury. “Priming” memory is stored in seemingly successfully repaired tubular cells in the absence of detectable persistent phenotypic changes, which may enhance a subsequent transcriptional response to the second stimulus. This type of memory may contribute to AKI-to-CKD transition through the cumulative effects of enhanced expression of profibrotic genes required for wound repair after recurrent AKI. Further understanding of epigenetic memory will identify therapeutic targets of future epigenetic intervention to prevent AKI-to-CKD transition.

## 1 Introduction

Epigenetics refers to the study of modifications of gene expression patterns that are not caused by alterations in DNA nucleotide sequences (Egger et al., 2004). The ability of the cells to retain and transmit changes in gene expression patterns induced by preceding stimuli encountered by daughter cells is described as epigenetic memory, which is conferred by epigenetic marks such as DNA methylation, histone modifications, histone variants, noncoding RNAs, and chromatin conformational changes (D'Urso and Brickner, 2014; Thiagalingam, 2020). Epigenetic memory is acquired during development or in response to environmental stimuli (D'Urso and Brickner, 2014). In addition to programmed epigenetic memory acquired for cell differentiation at each stage of development (Thiagalingam, 2020), epigenetic alterations that differentiated cells acquire when exposed to subsequent environmental stimuli are also stored as epigenetic memory (Wanner and Bechtel-Walz, 2017), which predisposes to late-onset diseases (Prachayasittikul et al., 2017). These acquired epigenetic memory events serve as promising therapeutic targets for diverse human diseases.

In the field of kidney diseases, the memory property of epigenetics is widely accepted as a contributor to “metabolic memory” in diabetic kidney disease (DKD) (Mimura et al., 2016; Natarajan, 2021; Zheng et al., 2021); a series of large-scale clinical studies show that former episodes of hyperglycemia pose a continuing risk of diabetic complications, even if the blood glucose level is subsequently maintained within normal limits (Holman et al., 2008; de Boer et al., 2011; de Boer, 2014; Nathan, 2014). Large-scale clinical trials with long-term follow-up periods (Miao et al., 2014; Chen et al., 2016; Chen et al., 2020) and various experimental models (El-Osta et al., 2008; Villeneuve et al., 2008; Villeneuve et al., 2010; Jia et al., 2019; Bansal et al., 2020) suggest that epigenetic alterations, including DNA methylation, histone modification, and noncoding RNAs, contribute to metabolic memory by promoting gene expression associated with inflammation and fibrosis. Similarly, in acute kidney injury (AKI)-to-chronic kidney disease (CKD) transition, a preceding episode of injury contributes to progression to chronic conditions, even after functional recovery from the initial injury. AKI is a frequently occurring disease, defined by a sudden loss of renal function, accompanied by factors including ischemia, sepsis, and chemotherapy using nephrotoxic drugs (Kellum et al., 2021). AKI was once recognized as a transient event, which resolves without sequelae. However, recent epidemiological and animal studies suggest a causal relationship between previous AKI episodes and subsequent progression to CKD (Coca et al., 2012; Tanaka et al., 2014; Basile et al., 2016; Nangaku et al., 2017; See et al., 2019), which is defined as persistent alterations in kidney structure or renal function, regardless of the nature of the initial injury, and whose common final pathological manifestation is tubulointerstitial fibrosis (Nangaku et al., 2017; Romagnani et al., 2017). Moreover, evidence indicates that this AKI-to-CKD transition is mediated by multiple mechanisms including epigenetic modifications (Zager, 2013; Nangaku et al., 2017; Ullah and Basile, 2019; Tanemoto and Mimura, 2022). However, its pathogenesis is insufficiently understood, particularly the detailed molecular mechanisms of the pathological contribution of such modifications.

While the prevalence of CKD and its subsequent progression to end-stage renal disease (ESRD), requiring renal replacement therapy, is increasing worldwide, there are no effective therapies for renal fibrosis (Jun and Lau, 2018). Evidence indicates that therapies targeting epigenetic mechanisms show promise, and epigenetic drugs tested in animal models of renal fibrosis have achieved positive effects (Fontecha-Barriuso et al., 2018; Guo et al., 2019; Li and Li, 2021; Tanemoto and Mimura, 2022). However, these drugs did not undergo clinical trials for kidney diseases, mainly because of their low specificity and a wide range of off-target effects such as the induction of global epigenetic alterations (Guo et al., 2019). In contrast, recent remarkable advances in epigenetic technology aim to overcome these weaknesses. For example, a recently developed CRISPR-Cas9-based method effectively offsets the broad off-target effect through the induction of locus-specific alterations of epigenetic marks of specific genes (Kato and Natarajan, 2019). Thus, this method activates renoprotective genes to attenuate renal fibrosis in a mouse model of kidney fibrosis (Xu et al., 2018). Moreover, approaches targeting noncoding RNAs also show promise because of recent technical advances (Kato and Natarajan, 2019). Chemically modified antisense oligonucleotides, such as locked nucleic acid (LNA)-modified anti-miRNAs, which inhibit specific miRNAs, are effective in mouse models of DKD (Kato and Natarajan, 2019). To take full advantage of these evolving technologies, acquiring detailed knowledge of the molecular mechanisms underlying AKI-to-CKD transition will better define potential therapeutic targets.

Here we focus on epigenetic memory in AKI-to-CKD transition: the epigenetic alterations induced by AKI that persist and contribute to the development of subsequent CKD. We review the most recent findings on the molecular basis of epigenetic memory and the pathophysiology of AKI-to-CKD transition. Accordingly, we present plausible hypothetical mechanisms of epigenetic memory that mediate this transition and the prospects for their potential therapeutic application.

## 2 Update on the pathophysiology of AKI-to-CKD transition

### 2.1 Major factors contributing to AKI-to-CKD transition: Renal hypoxia as a key player

Renal homeostasis is maintained by multiple types of differentiated cells with specific functions that compose the kidneys (Sato et al., 2020). AKI-to-CKD transition is therefore mediated by the interplay among tubular epithelial cells, endothelial cells, pericytes, inflammatory cells, and myofibroblasts (Guzzi et al., 2019; Tanemoto and Mimura, 2022). Tubular injury leading to nephron loss and maladaptive tubular repair, endothelial dysfunction with vascular rarefaction, interstitial inflammation, and fibrosis represent the main pathologies that contribute to AKI-to-CKD transition; and renal hypoxia, which interacts with these pathologies, is an important factor for disease progression (Tanaka et al., 2014; Ullah and Basile, 2019; Tanemoto and Mimura, 2022).

The kidney is physiologically prone to hypoxia because of its characteristic vascular structure and high metabolic demand (Sugahara et al., 2020). The peritubular capillaries perfusing the renal tubules possess a diffusional oxygen shunt between closely parallel arterial and venous vessels, which limits oxygen extraction by the kidney by as much as 10%; therefore, the kidney is intrinsically vulnerable to further hypoxic stress (Nangaku, 2006; Mimura and Nangaku, 2010; Nangaku et al., 2017). Moreover, the high energy demand of proximal tubular cells required for solute transport mainly relies on aerobic respiration, which imparts susceptibility to oxygen deprivation (Evans et al., 2014; Sugahara et al., 2020). Proximal tubular cells are therefore very vulnerable to ischemic injury, which is the leading cause of AKI, and are considered the primary target of AKI (Chevalier, 2016; Schiessl, 2020).

Renal epithelial cells possess remarkable regenerative capacity, which enables them to completely recover from mild injury with subsequent resolution of renal perfusion (Kramann et al., 2015; Schiessl, 2020). However, because of the limited capacity of tubular cell proliferation, increased severity of AKI leads to maladaptive tubular repair, which predisposes to fibrosis and CKD (Basile et al., 2016; Ullah and Basile, 2019). Indeed, in murine ischemia-reperfusion injury (IRI) models representing ischemic AKI, a longer duration of ischemia inflicts severe initial tubular damage and increased fibrosis 4 weeks after reperfusion (Dong et al., 2019). Injured tubular epithelial cells undergo phenotypic changes to produce proinflammatory and profibrotic substances (Grgic et al., 2012; Liu et al., 2018). Thus, evidence indicates that epigenetic alterations, as well as maladaptive tubular repair, contribute to this process (Zager, 2013; Nangaku et al., 2017). The current view of the detailed mechanisms of tubular injury and repair are reviewed in Section 2.3.

The limited regenerative capacity of the renal microvasculature, in contrast to renal tubules, explains the AKI-induced reduction in the number of capillaries, called “capillary rarefaction,” which results in chronic renal hypoxia, contributing to CKD progression (Basile, 2004; Ullah and Basile, 2019). The underlying mechanisms of capillary rarefaction may involve decreased expression of the angiogenic factor vascular endothelial growth factor (VEGF) from tubular epithelial cells and detachment of pericytes localized close to endothelial cells that maintain vascular stability (Tanaka et al., 2014; Tanemoto and Mimura, 2022).

Subsequent renal hypoxia induces expression of proinflammatory and profibrotic gene of tubular epithelial cells through epigenetic alterations, which is called “hypoxic memory” (reviewed Section 3.1) (Nangaku et al., 2017; Tanaka, 2018). Hypoxia also activates inflammatory cells, including resident and infiltrating immune cells such as macrophages and neutrophils, which contribute to tissue regeneration and scar formation, leading to tubulointerstitial fibrosis (Anders, 2014; Guzzi et al., 2019).

Myofibroblasts are activated forms of fibroblasts that produce large amounts of extracellular matrix (ECM) that contributes to tissue fibrosis formation (Jun and Lau, 2018). These cells are predominantly derived from resident fibroblasts and pericytes (Kuppe et al., 2021) (other origins include bone marrow-derived macrophages (Tang et al., 2019; Chen J. et al., 2022) and endothelial-to-mesenchymal transition (Chen Y. et al., 2022)), and their function is influenced by profibrotic signals emanating from tubular cells and inflammatory cells (Ullah and Basile, 2019). Tubulointerstitial fibrosis aggravates renal hypoxia through further loss of capillaries and the increasing physical distance between resident tubular cells and capillaries. This vicious cycle of renal hypoxia and tubulointerstitial fibrosis accelerates subsequent progression of CKD (Nangaku, 2006; Tanemoto and Mimura, 2022).

Renal hypoxia, as described above, contributes to the pathophysiology of AKI-to-CKD transition. Indeed, renal hypoxia occurs in various forms of CKD (Sugahara et al., 2020). For example, a prospective study shows that oxygenation of renal tissue of patients with CKD, detected using blood oxygenation level-dependent magnetic resonance imaging (BOLD-MRI), is low in the renal cortex, which serves as an independent predictor of deteriorating renal function (Pruijm et al., 2018). Further, renal hypoxia in rats induced by dinitrophenol (a mitochondrial uncoupler that increases oxygen consumption) for 30 days suggests that renal tissue hypoxia alone may serve as an efficient trigger of the development of nephropathy (Friederich-Persson et al., 2013). These studies confirmed the pathologically significant contribution of renal hypoxia to the development of CKD.

### 2.2 Metabolic reprogramming during AKI-to-CKD transition

Recent studies suggest “metabolic reprogramming” of tubular epithelial cells is also an important factor that contributes to the AKI-to-CKD transition (Gomez et al., 2017; Zhu et al., 2022). As an adaptive response to hypoxic microenvironments, cancer cells shift energy sources from mitochondrial fatty acid β-oxidation (FAO) to glycolysis, which is known as the Warburg effect (Sun et al., 2018; Zhu et al., 2022). As mentioned in the previous section, tubular epithelial cells have high energy demands rendering them susceptible to AKI. To compensate for the lack of energy production resulting from hypoxia and mitochondrial dysfunction during AKI, similar to cancer cells, tubular epithelial cells undergo metabolic reprogramming, shifting away from FAO to glycolysis (Faivre et al., 2021; Tang et al., 2021). This process is considered a protective process for surviving tubular epithelial cells during the repair phase of AKI; besides restoring ATP production, enhanced glycolysis following acute injury contributes to fibroblast activation by generating lactate (the final product of glycolysis), which may promote wound healing and tubular regeneration as a protective inflammatory response (Gomez et al., 2017; Shen et al., 2020; Li et al., 2021). However, this metabolic reprogramming is a double-edged sword with respect to renal prognosis; the persistent shutdown of FAO and enhanced glycolysis in tubular epithelial cells contributes to the transition to CKD accompanied by chronic inflammation and lipid accumulation (Schaub et al., 2021; Zhu et al., 2022). Kang et al. showed that fibrotic kidneys from humans and mice exhibit lower expression of enzymes and regulators involved in FAO (Kang et al., 2015). In particular, they demonstrated that decreased FAO alone is sufficient to induce a phenotypic change that promotes fibrosis in tubular epithelial cells, whereas genetic or pharmacologic replenishment of FAO protects mice from kidney fibrosis (Kang et al., 2015). Lipid accumulation in nonadipose tissue results in lipotoxicity, including cell dysfunction and necrosis (Zhu et al., 2022). Decreased FAO results in the reduced use of free fatty acids in tubular epithelial cells, which promotes lipid accumulation and tubulointerstitial fibrosis (Jang et al., 2020; Zhu et al., 2022).

Studies suggest that successful switching metabolism from glycolysis to FAO after AKI is an important checkpoint for determining whether to progress to CKD. Identifying the mechanisms of maladaptive persistence of metabolic reprogramming following AKI even after tubular epithelial cells are successfully repaired, including the presence or absence of epigenetic memory, requires further investigation.

### 2.3 Current understanding of tubular repair

#### 2.3.1 Tubular epithelial cells may act as “holders” of epigenetic memory

The ischemia-reperfusion injury (IRI) model is a validated animal model of AKI-to-CKD transition that induces transient renal ischemia through clamping renal arteries, which generates tubulointerstitial fibrosis weeks later (Mimura et al., 2018).

Liu et al. conducted RNA sequencing (RNA-seq) of whole kidney using murine IRI models, from 2 h to 12 months following reperfusion, which reveal dynamic changes in gene expression, characterized by several functionally related temporal-specific patterns of gene expression, including tubular injury and repair, fibrosis, and the inflammatory response (Liu et al., 2017). These transcriptomic dynamics during the transition from AKI to CKD are mediated by cell types that compose the kidney. Although each cell type serves as a “holder” of epigenetic memory, the present review focuses on tubular epithelial cells representing the major cell population of the kidney, which is most susceptible to hypoxic stress and undergoes AKI-induced transitional phenotypic changes. Such cells significantly contribute to the AKI-to-CKD transition, possibly influenced by persistent epigenetic alterations induced by the preceding AKI episode. The following summarizes the current view of the molecular mechanism of tubular repair (Little and Humphreys, 2022; Naved et al., 2022).

#### 2.3.2 Tubular epithelial cells proliferate through dedifferentiation in response to tubular injury

Proximal tubular cells are the most sensitive to AKI among renal cell populations. Their regenerative capacity is limited, and the success or failure of tubular repair may depend on the intensity of an injury (Takaori et al., 2016). Acute injury in proximal tubules (PTs) peaks 48 h after injury, followed by tubular proliferation (Chang-Panesso et al., 2019). Evidence strongly supports the conclusion that resident tubular epithelial cells serve as the primary cellular source that replenishes lost tubular cells (Humphreys et al., 2008; Schiessl, 2020); however, the identity of intratubular cells responsible for the repair is still controversial (Schiessl, 2020; Little and Humphreys, 2022).

Convincing evidence suggests that surviving proximal tubular epithelial cells dedifferentiate and re-enter the cell cycle, undergoing proliferative expansion required for reconstructing damaged tubules, followed by proliferation and redifferentiation (Humphreys et al., 2011; Kusaba et al., 2014; Ullah and Basile, 2019; Little and Humphreys, 2022). To gain proliferative capacity, dedifferentiated tubular cells lose certain markers of terminal differentiation including cadherin, and gain vimentin expression and certain markers of development and injury, including kidney-injury molecule-1 (*Kim-1*) (Chang-Panesso and Humphreys, 2017). In contrast, a fixed population of *Pax2* ^*+*^ */Kim-1* ^*-*^ intratubular proximal tubular progenitor cells undergo clonal expansion that mediates repair, serving as a model of the progenitor cells responsible for tubular repair (Lazzeri et al., 2018). However, a recent genetic lineage tracing analysis of dedifferentiated tubular cells expressing *Kim-1* after injury shows the proliferative expansion of *Kim-1* ^*+*^ dedifferentiated cells during tubular repair, suggesting that the proliferating cells after injury express the injury marker *Kim-1*, which does not support the contribution of *Pax2* ^*+*^ */Kim-1* ^*-*^ progenitors to tubular repair (Chang-Panesso et al., 2019; Little and Humphreys, 2022).

#### 2.3.3 A distinct subset of proximal tubular cells acquires a new phenotype after AKI

Recent single-cell analysis of AKI-to-CKD transition reveals distinct states of tubular epithelial cells after acute injury. The “failed repair” cell state characterized by the molecular markers *Vcam1* ^*+*^ */Ccl2* ^*+*^ was identified using time-course RNA-seq subsequent to IRI in mouse models, which is called the “failed repair proximal tubular cell” (FR-PTC) (Kirita et al., 2020; Gerhardt et al., 2021). These “failed repair” cells represent 5%–10% of the cell population of the proximal tubule after AKI and are also present in humans after AKI (Little and Humphreys, 2022). Such cells express activated NF-κB and secrete proinflammatory and profibrotic cytokines, suggesting that they mediate AKI-to-CKD transition (Little and Humphreys, 2022). Further, joint profiling of chromatin accessibility and gene expression of healthy human kidneys (specimens acquired after mass nephrectomy) using single-nucleus RNA-seq and single-nucleus ATAC-seq (assay for transposase-accessible chromatin with high-throughput sequencing) captures open chromatin sites (Muto et al., 2021). This study reveals a small subset of proximal tubular cells, characterized by *VCAM1* expression and NF-κB activity, without a preceding episode of AKI, are transcriptionally similar to “failed repair” cells (Muto et al., 2021; Little and Humphreys, 2022). This subset of cells may also contribute to renal fibrosis.

### 2.4 Fibrosis as a proregenerative factor in the early phase of kidney injury

#### 2.4.1 Tubular repair after AKI is mediated by surrounding fibroblasts through mechanical support and paracrine signaling

**FIGURE 1:**
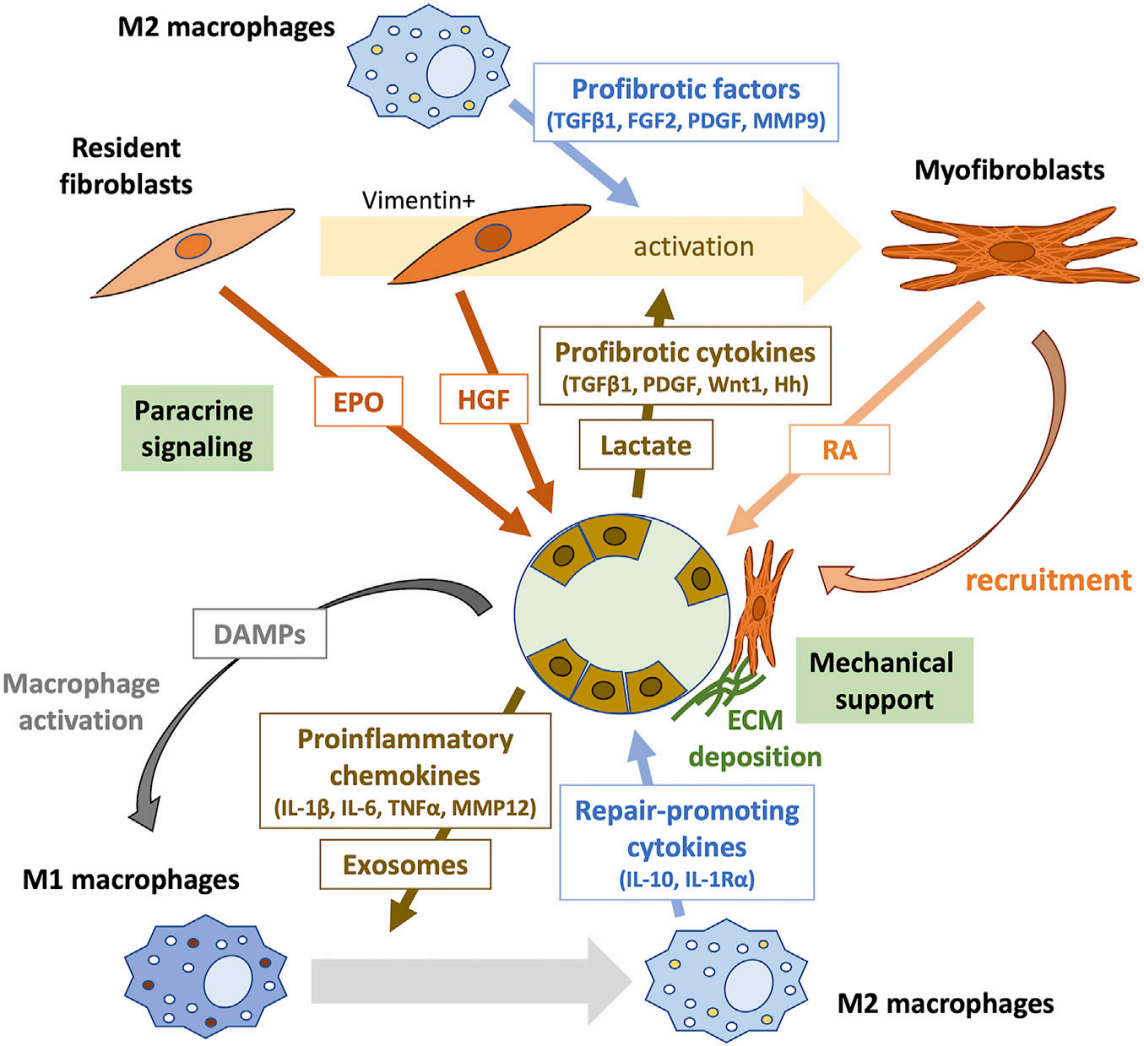
Tubule-interstitial crosstalk during the early phase of kidney repair. The crosstalk between tubular epithelial cells, inflammatory cells, and interstitial fibroblasts plays an important role in kidney repair. Surviving proximal tubular epithelial cells undergo dedifferentiation to proliferate and repopulate the tubular basement membrane. Tubular cell death initiates inflammation by releasing DAMPs, which activate pattern recognition receptors (PRRs) on macrophages. Damaged dedifferentiated tubular epithelial cells acquire a proinflammatory phenotype and secret various chemokines that recruit immune cells to the injury site. They may also communicate with immune cells through exosomes containing signaling molecules. During kidney repair, proinflammatory (M1) macrophages change phenotype to M2 and produce repair-promoting cytokines, such as IL-10 and IL-1Rα. Furthermore, M2 macrophages secrete profibrotic factors, including TGFβ1, FGF2, PDGF, and MMP9, which induce the transformation of surrounding resident fibroblasts into activated myofibroblasts. Profibrotic cytokines, such as TGFβ1, PDGF, Wnt1, and hedgehog (Hh), and lactate produced by injured tubular epithelial cells also contribute to fibroblast activation. Resident fibroblasts in the renal interstitium promote tubular repair through paracrine signaling. EPO produced by PDGFR-β^+^ fibroblasts mediate protective effects against tubular injury during the early phase of kidney repair. Early activation of fibroblasts after AKI results in the secretion of the proregenerative cytokine HGF. Activated myofibroblasts provide mechanical support by forming tight junctions, produce the ECM, and promote tubular repair through intercellular contact signaling and paracrine signaling of RA. DAMPs, damage-related molecular patterns; IL, interleukin; TNFα, tumor necrosis factor-α; MMP, metalloproteinase; TGFβ1, transforming growth factor-β1; FGF2, fibroblast growth factor-2; PDGF, platelet-derived growth factor; MMP9, metalloproteinase-9; Hh, hedgehog; EPO, erythropoietin; HGF, hepatocyte growth factor; RA, retinoic acid.

Epigenetic memory affecting the acquisition of a pathogenic phenotype, such as proinflammatory and profibrotic phenotypes involved in the pathophysiology of AKI-to-CKD transition, if present as in DKD, may serve as promising therapeutic targets. Whereas renal fibrosis represents the hallmark of CKD, emerging evidence suggests a proregenerative effect of fibrosis on kidney repair following AKI, particularly during the early phase (Schiessl, 2020). Here we briefly review tubule-interstitial crosstalk in the repair phase and recent evidence indicating the possible beneficial effects of fibrosis on tubular repair (Figure 1). The importance of timely therapeutic intervention will be discussed in the next section.

Tubular remodeling following injury is mediated by a significant capacity to recover tubular cells (reviewed above in Section 2.3.2) and through the interaction between tubular and interstitial cells (Qi and Yang, 2018; Schiessl, 2020). In the early stage of AKI, necrotic and damaged tubular cells release damage-related molecular patterns (DAMPs) and recruited neutrophils generate reactive oxygen species (ROS), which promote macrophage activation (Fu et al., 2022). In the first 48 h after AKI, proinflammatory (M1) macrophages are recruited to the kidney, which produce cytokines for robust inflammation. In the later repair phase, they undergo a phenotypic change to noninflammatory (M2) macrophages, which contributes to fibroblast activation by producing profibrotic factors, including transforming growth factor-β1 (TGFβ1), fibroblast growth factor-2 (FGF2), platelet-derived growth factor (PDGF), and metalloproteinase-9 (MMP9) (Lee S. et al., 2011; Sato and Yanagita, 2018; Fu et al., 2022). Further, tubular injury results in the release of cytokines from the injured tubular epithelial cells, such as TGFβ1, PDGF, Wnt1, and hedgehog (Hh), which activate the surrounding fibroblasts in a paracrine manner (Maarouf et al., 2016; Qi and Yang, 2018; Schiessl, 2020). Lactate generated from glycolysis in injured tubular epithelial cells also induces fibroblast activation and proliferation (Shen et al., 2020).

Subsequently, activated myofibroblasts are locally recruited around regenerating tubules, which act as a structural support through the formation of a tight arrangement surrounding the tubular basal membrane (Fujigaki et al., 2005; Schiessl, 2020). This myofibroblast recruitment is reversible after successful tubular regeneration, suggesting that this local fibrotic process supports the recovery of the tubules (Kaissling et al., 2013).

Moreover, recent studies suggest that interstitial fibroblasts contribute to tubular repair through paracrine signaling (Shi et al., 2018; Zhou et al., 2018; Nakamura et al., 2019; Schiessl, 2020). For example, Zhou et al. found that a fibroblast-specific deletion of the gene encoding β-catenin in mice leads to renoprotective effects against IRI by increasing the secretion of hepatocyte growth factor (HGF) from fibroblasts, which enhances HGF/c-met signaling in tubular cells and promotes their survival and proliferation (Zhou et al., 2018). They also showed that tubule-derived sonic hedgehog (Shh) triggered by tubular injury induces early activation of fibroblasts occurring as early as 1 h after injury; the resulting vimentin-positive activated fibroblasts produce and secret HGF, promoting kidney repair and regeneration (Zhou et al., 2019). Shi et al. found that genetic deletion of the gene encoding the erythropoietin receptor (EpoR) in tubular epithelial cells increases renal susceptibility to IRI in mice (Shi et al., 2018). EPO is mainly synthesized by PDGFR-β^+^ fibroblasts and is released to stimulate adjacent tubular cells (Gerl et al., 2016). Thus, the disruption of EPO/EpoR signaling may inhibit tubule-interstitial crosstalk beneficial to tubular repair (Shi et al., 2018; Schiessl, 2020). Notably, constitutive hyperstimulation of EpoR signaling disturbs capillary formation and exacerbates tubulointerstitial fibrosis at the subacute stage (day 14 after IRI), suggesting that EPO/EpoR signaling may exert the opposite effects on kidney outcomes at different times subsequent to injury and recovery (Shi et al., 2018; Schiessl, 2020). Nakamura et al. performed using unilateral ureteral obstruction (UUO) of mice to show that diphtheria toxin (DT)-mediated selective disruption of protein synthesis of renal fibroblasts, which attenuates tubular proliferation in fibrotic kidneys accompanied by downregulation of the retinoic acid (RA) signaling pathway (Nakamura et al., 2019). Upon injury, when proximal tubules fail to express retinaldehyde dehydrogenase 2 (RALDH2), the essential enzyme in RA biosynthesis, RALDH2 activity is compensated by increased expression in myofibroblasts, which is inhibited in DT-treated mice, resulting in attenuated tubular regeneration. These results suggest the beneficial effect of fibrosis on tubular repair mediated by RA signaling (Nakamura et al., 2019).

#### 2.4.2 Kidney repair during the early phase after AKI: Association with enhancer dynamics regulated by epigenetic alterations

A recent study targeting the enhancer and super-enhancer landscapes of kidney cortex samples, most of which consists of tubular epithelial cells, subsequent to AKI, identified key transcription factors (TFs) associated with kidney repair, providing insight into the appropriate timing of antifibrotic therapeutic intervention (Wilflingseder et al., 2020). Super-enhancers (SEs) are clusters of enhancers occupied by an extremely high density of coactivators, leading to increased levels of transcription compared to typical enhancers (Sabari et al., 2018). Enhancers and SEs primarily define the frequency of stochastic transcriptional bursting, a key parameter that generates cell-type specific gene expression, which raises the possibility of the engagement of enhancer and SE dynamics in the phenotypic change in response to kidney injury (Whyte et al., 2013; Fukaya et al., 2016; Larsson et al., 2019). Wilflingseder et al. performed chromatin immunoprecipitation sequencing (ChIP-seq) and sample-matched RNA-seq analyses of uninjured and repairing mouse kidneys 2 days after AKI, revealing that kidney injury leads to genome-wide alterations in the enhancer and SE repertoires characterized by upregulation of their regulatory, injury-responsive genes during kidney repair (Wilflingseder et al., 2020). Hepatocyte nuclear factor 4 alpha (HNF4A), glucocorticoid receptor (GR), signal transducer and activator of transcription (STAT) 3, and STAT5 were identified as TFs that specifically bind enhancer and SE sites during kidney repair (Wilflingseder et al., 2020). This study used the bromodomain and external domain (BET) inhibitor JQ-1 to block enhancer functions before or soon after AKI through inhibition of bromodomain containing protein 4 (BRD4). This treatment affects repair responses leading to impaired recovery. Further, the administration of daily JQ-1 initiated within 48 h post-IRI impairs response and causes a higher mortality rate after IRI; in contrast, daily JQ-1 administered after day-3 post-IRI does not increase the mortality rate compared with that the vehicle-treated group (Wilflingseder et al., 2020). Further, in mouse models of kidney fibrosis, JQ-1 treatment starting on day-7 after aristolochic acid (AA) injection and on day-10 after UUO, each alleviate subsequent development of kidney fibrosis, suggesting that the effect of BET inhibition on kidneys differ depending on the timing, and importantly, inhibit the development of fibrosis administered later after injury (Wilflingseder et al., 2020).

In this chapter, we emphasize the possible protective role of fibrosis during the early stage of kidney injury. In contrast, if these proregenerative responses maladaptively persist, those responses may switch upon subsequent progression of tubulointerstitial fibrosis. Further, epigenetic alterations are involved in gene expression dynamics after AKI through enhancer regulation, and intervention in the action of these factors may modulate postinjury responses. Epigenetic alterations trigger dynamic phenotypic changes by regulating enhancer and SE activity. If epigenetic memory induces maladaptive extension of the normal wound healing process, it may serve as a promising therapeutic target.

## 3 Epigenetic memory in AKI-to-CKD transition: Persistent epigenetic alterations identified in AKI-to-CKD transition

### 3.1 Hypoxia-induced epigenetic alterations are retained in cells as “hypoxic memory.”

Our group focuses on renal hypoxia as a common pathway driving the transition of AKI to CKD. We identified epigenetic alterations that may contribute to the progression of fibrosis induced by hypoxic stimuli.

Hypoxia-inducible factor (HIF) is a master TF that mediates the adaptive response to hypoxia through the activation of target genes harboring hypoxia-responsive elements (HREs) within their regulatory domains (Nangaku et al., 2013). Signaling induced by HIF targets >100 genes, including those that regulate hematopoiesis, angiogenesis, and anaerobic metabolism (Mole et al., 2009; Tanaka et al., 2014). Similar to the functions of other TFs, HIF modulates chromatin states of the regulatory domains of their target genes through the recruitment of epigenetic modifiers such as histone modifying enzymes and chromatin remodeling proteins (Arany et al., 1996; Lee et al., 2010; Lee J. S. et al., 2011; Mimura et al., 2013; Perez-Perri et al., 2016; Luo and Wang, 2018).

The temporal profile of gene expression under hypoxia in human umbilical vein endothelial cells (HUVECs), as revealed by analysis of a DNA microarray, detected early hypoxia-responsive genes, which are functionally associated with glycolysis, including the gene encoding solute carrier family 2A3: SLC2A3 (GLUT3) (Mimura et al., 2012). We found that HIF-1 functions as an enhancer of *SLC2A3* transcription through its interaction with lysine-specific demethylase 3A (KDM3A), a HIF-1-induced protein that demethylates repressive dimethylated H3 lysine 9 (H3K9me2) (Mimura et al., 2012). Further, the regulatory mechanism of *SLC2A3* is accompanied by a chromosomal conformational change, as revealed by the chromatin conformation capture (3C) assay. Under normoxic conditions, the 35-kb upstream region of the *SLC2A3* promoter (enhancer 1) is structurally close to the transcriptional start site (TSS) through the binding of HIF-1 located in both sites, forming a loop, which is lost when HIF-1 expression is inhibited by a specific small interfering RNA (siRNA). In contrast, under hypoxic conditions, HIF-1 additionally binds to the 24-kb upstream region of the *SLC2A3* promoter (enhancer 2), and KDM3A is recruited via the interaction with HIF-1 located in the TSS, enhancer 1, and 2 with two loops, enabling robust expression of *SLC2A3*, which demonstrates that HIF-1-dependent epigenetic alterations in chromatin conformation regulate downstream gene expression (Mimura et al., 2012). Moreover, KDM3A promotes fibrosis by activating profibrotic *Timp1* transcription in cardiomyocytes, and inhibition of KDM3A is suppressed by cardiac fibrosis *in vivo* (Zhang et al., 2018), suggesting that KDM3A may mediate fibrogenesis in the kidney.

Genome-wide analysis of HIF-1 binding sites of cultured tubular cells exposed to a hypoxic environment using RNA-seq and ChIP-seq identified 44 long noncoding RNAs (lncRNAs) that are upregulated under hypoxia in multiple tubular cell lines (Mimura et al., 2014; Mimura et al., 2017). For example, DARS-AS1 (aspartyl-tRNA synthetase anti-sense 1) is a lncRNAs that is specifically upregulated under hypoxia through the binding of HIF-1 in the promoter of the gene encoding DARS-AS1 harboring two HREs (Mimura et al., 2017). Further, DARS-AS1 inhibits apoptosis in renal tubular cells, suggesting its ability to inhibit renal fibrosis (Mimura et al., 2017).

As described in Section 1, cells store epigenetic alterations induced by preceding stimuli as epigenetic memory. In AKI-to-CKD transition, hypoxia-induced epigenetic changes acquired during the initial AKI episode are retained in the cells and exert long-term effect, termed “hypoxic memory” (Nangaku et al., 2017; Tanaka, 2018). This specifically refers to “persistent” epigenetic alterations that contribute to the progression of CKD subsequent to the initial AKI-induced hypoxia.

Increasing evidence has demonstrated that epigenetic alterations are involved in the pathophysiology of AKI-to-CKD transition (Fontecha-Barriuso et al., 2018; Guo et al., 2019; Li and Li, 2021; Tanemoto and Mimura, 2022); however, most of them, including those mentioned in this section, report how epigenetic alterations are induced as a result of kidney injury, rather than the persistence of epigenetic alterations. Here, we would like to focus on the effects of persistence of epigenetic alterations induced by AKI on the subsequent progression to CKD. Epigenetic changes identified at a specific timepoint may be merely a consequence of a transcriptional response. In contrast, persistent epigenetic changes induced by AKI may “actively” contribute to downstream phenotypic changes, which has attracted our interest. In this review, we will focus on persistent epigenetic alterations, or epigenetic memory, which contribute to the pathogenesis of AKI-to-CKD transition.

### 3.2 Examples of “epigenetic memory” induced by ischemic injury that persists during AKI-to-CKD transition

There are several studies focusing on the persistence of epigenetic alterations during AKI-to-CKD transition. Most of the animal models use IRI model because it is the most common cause of AKI.

Zager et al. found that a single IRI in mice induces upregulation of histone-modifying enzymes (SET1 and BRG1), and elevation of gene-activating histone modifications (H3K4me3 and histone variant H2A.Z) at the promoters of the genes encoding the proinflammatory monocyte chemoattractant protein 1 (MCP1), the profibrotic TGFβ1, and collagen III (Zager and Johnson, 2009). These events induce the transcriptional upregulation of these genes as well as renal histological fibrosis (Zager and Johnson, 2009). Further, such transcriptional upregulation is maintained for 3 weeks, accompanied by persistent gene-activating histone modifications at the promoters of those genes (Zager et al., 2011), suggesting persistent epigenetic “memory” of ischemic injury. Moreover, to determine whether changes in histone modifications caused or were a consequence of increased transcription, the kinetics of elevation of histone modification and gene transcription were determined using a mouse model of rhabdomyolysis-induced AKI (Zager and Johnson, 2010). This study found that the increase in histone modifications (H3K4me3 and H2A.Z) precede elevated messenger RNA (mRNA) levels, which were not attributed to mRNA stabilization, suggesting that gene-activating histone modifications opened the chromatin structure to enhance transcription (Zager and Johnson, 2010). The authors designated cellular adaptation that reprograms the cellular response after AKI as “biologic memory”; and the persistent epigenetic alterations identified above are described as “AKI-initiated epigenetic remodeling,” which among other factors, contribute to “biologic memory” (Zager, 2013). Further, these events are considered candidates for “hypoxic memory”. The salient point is that regardless of whether they are directly attributed to hypoxia, epigenetic alterations, possibly induced by preceding AKI, are retained in cells with altered gene expression profiles.

Further, changes in DNA methylation patterns induced by IRI can be also epigenetic memory contributing to AKI-to-CKD transition. Several underlying mechanisms that IRI affects DNA methylation have been proposed: 1. IRI-induced oxidative stress causes DNA lesions, which affects the binding of DNA methyltransferases (DNMTs) and methyl-binding proteins; 2. Ten-eleven translocation (TET) enzymes (responsible for active DNA demethylation) is arrested during ischemia because of oxygen depletion; and 3. Hypoxia during ischemia directly modulates the expression of DNMTs and TET enzymes (Franco et al., 2008; Tahiliani et al., 2009; Huang et al., 2012; Watson et al., 2014; Heylen et al., 2016).

Zhao et al. conducted the genome-wide DNA methylation analysis 24 h and 7 days after IRI in murine models of IRI to evaluate the role of DNA methylation in AKI-to-CKD transition (Zhao et al., 2017). Eighteen genes with persistent alterations in DNA methylation induced by IRI for 7 days, as well as persistent changes in gene expression, were identified, suggesting that persistent promoter methylation contributes to the pathophysiology of AKI-to-CKD transition (Zhao et al., 2017). Others analyzed DNA methylation and gene expression in a rat model of IRI, focusing on HIF1α/VEGF signaling at 1, 2, 3, and 4 months after IRI (Sanchez-Navarro et al., 2021). Rats that experience IRI exhibit a persistent reduction in global DNA methylation during the entire experiment; and the reduction of HIF1α/VEGF signaling triggered after IRI, which may trigger renal hypoxia, is accompanied by persistent hypermethylation at the HIF-1α binding site of the *Vegfα* promoter throughout the observational periods (Sanchez-Navarro et al., 2021). Thus, DNA methylation, similar to its role in DKD, is a strong candidate for epigenetic memory in AKI-to-CKD transition.

### 3.3 Epigenetic memory in other models of AKI-to-CKD transition

In kidney transplantation, chronic allograft injury is associated with IRI, which cannot be avoided during transplantation, and is a condition similar to AKI-to-CKD transition (Cavaillé-Coll et al., 2013). Indeed, each additional hour of cold ischemia time (during cold storage before transplantation) increases the risk of graft failure and death (Debout et al., 2015). Several studies have suggested that epigenetic alterations induced by cold ischemia during transplantation remain afterward and contribute to disease progression to CKD (Xiang et al., 2022). Pratt et al. showed that IRI during transplantation (24 h of cold ischemia and a subsequent 2 h of reperfusion) induces DNA demethylation within the interferon γ (IFNγ)-responsive element in the promoter region of the complement component 3 (*C3*) gene in rat kidney, thus implicating local C3 synthesis by tubular epithelial cells (Pratt et al., 2006); subsequently, they found that demethylation of putative regulatory sites in the *C3* promoter in both ischemic control group (48 h of reperfusion) and chronic nephropathy group (6 months after transplantation), suggesting that aberrant DNA demethylation persists throughout disease progression to chronic allograft injury (Parker et al., 2008). Heylen et al. profiled genome-wide DNA methylation in kidney allograft biopsy specimens at multiple timepoints: 1) at procurement (preischemia), 2) after implantation and reperfusion (postischemia), and 3) 3 or 12 months after transplant in a longitudinal cohort (Heylen et al., 2018). The results indicated that cold ischemia induces DNA hypermethylation of allografts through reduced TET activity, which preferentially decreases the expression of genes involved in the negative regulation of kidney fibrosis and apoptosis. Notably, the observed hypermethylation changes in CpG islands induced by cold ischemia were stable at 3 and 12 months after transplantation (Heylen et al., 2018), suggesting that they represent epigenetic memory that persistently alters the regulation of gene expression and contributes to disease progression to chronic allograft injury.

Sepsis is a fatal multiple organ dysfunction that results from a systemic inflammatory response induced by severe infection (Singer et al., 2016; Qiao and Cui, 2022). Sepsis-associated AKI (SA-AKI), defined as AKI involved in sepsis or septic shock, is common in the intensive care unit (ICU), where SA-AKI accounts for nearly half of all patients with AKI (Sun et al., 2019). SA-AKI may occur in the absence of renal hypoperfusion and global renal blood flow in SA-AKI may be normal or increased, suggesting that IRI is not the only contributor to SA-AKI (Peerapornratana et al., 2019; Qiao and Cui, 2022). The pathogenesis of SA-AKI is considered a synergistic interaction of several events, including inflammation, microcirculatory dysfunction (redistribution of blood flow and increased shunting), and metabolic reprogramming (Peerapornratana et al., 2019; Qiao and Cui, 2022). Indeed, Tran et al. performed microarray sequencing of kidney-derived RNA from mice at baseline and after lipopolysaccharide (LPS) administration (a modeling method for SA-AKI) and found that genes involved in oxidative phosphorylation, including the mitochondrial biogenesis regulator *PGC-1α*, are suppressed during SA-AKI and reactivated following the recovery of renal function, indicating that mitochondrial dysfunction is a major contributor to SA-AKI (Tran et al., 2011). With respect to epigenomics, Binnie et al. conducted a genome-wide DNA methylation analysis of whole blood samples from septic and nonseptic critically ill patients and identified a total of 668 differentially methylated regions (DMRs) corresponding to 443 genes, including those functionally involved in sepsis (Binnie et al., 2020). Evidence indicates that epigenetic interventions in AKI, including histone deacetylase (HDAC) inhibitors, are effective (Zhang et al., 2020); however, most target the improvement of AKI. Studies of epigenetic alterations using AKI-to-CKD transition as an outcome, including those focused on persistence, remain scarce. On the other hand, in sepsis, prior endotoxin exposure enhances the transcriptional response to subsequent septic stimuli, and epigenetic alterations are involved in this phenomenon. The contribution of tubular epithelial cells to this phenomenon has also been suggested, which is discussed in detail in Section 5.3.3.

Drug-induced nephrotoxicity is also one of the most common clinical causes of AKI and accounts for approximately 20% of all cases (Uchino et al., 2005; Sharp et al., 2016; Guo et al., 2017). Cisplatin is a widely used and highly effective platinum-based anticancer drug; however, its adverse nephrotoxic effects induce AKI in one-third of the patients receiving this drug, which limits the overall dose and efficacy (Sharp et al., 2016; Yamashita et al., 2021). Nuclear DNA damage plays a major role in cisplatin-induced nephrotoxicity. Cisplatin is absorbed in proximal tubular epithelial cells through organic cation transporters (OTCs) and binds and induces DNA damage. Severe DNA damage results in irreversible injury and apoptosis of proximal tubular epithelial cells (Pabla and Dong, 2008; Yamashita et al., 2021). Endoplasmic reticulum (ER) stress, oxidative stress, mitochondrial apoptosis, and infiltration of inflammatory cells are also involved in cisplatin-induced AKI (Huang et al., 2022). Guo et al. performed a genome-wide DNA methylation analysis of mouse kidneys after 3 days after administration of cisplatin or saline and identified 215 DMRs between cisplatin-treated kidneys and the control and 15 protein-coding genes with DMRs in the promoter or promoter-regulatory regions (Guo et al., 2017). They found that interferon regulatory factor 8 (*Irf8*) is hypomethylated in cisplatin-induced AKI, accompanied by a marked induction of Irf8, which contributes to the pathogenesis of cisplatin-induced AKI (Guo et al., 2017). However, there are no recent studies examining the persistence of such epigenetic alterations induced by cisplatin that contribute to AKI-to-CKD transition. Recently, Yamashita et al. demonstrated that repeated low dose cisplatin (established as a model of AKI-to-CKD transition) induce active proliferation of proximal tubular epithelial cells and thereby accumulates DNA damage (Yamashita et al., 2021). They identified a population of proximal tubular cells expressing *Vcam1* after repeated cisplatin administration, suggesting the induction of failed-repair proximal tubular cells with a proinflammatory phenotype identified in single-cell analysis using mouse IRI models (mentioned in Section 2.3.3) (Kirita et al., 2020; Yamashita et al., 2021). The possibility of the involvement of epigenetic memory in the formation of this specific cell cluster will be discussed in Section 6.

## 4 The molecular basis of epigenetic memory: Update on transcriptional regulation

In the previous chapters, which describe the latest findings of phenotypic changes in tubular epithelial cells during AKI-to-CKD transition, we mainly focus on epigenetic memory that continuously activates or inhibits the regulation of gene expression. However, this is not the only possible pattern that explains the basis of the influence of epigenetic memory on the progression of AKI-to-CKD transition. “Transcriptional memory,” the subject of numerous studies of epigenetic memory, refers to epigenetic changes induced by environmental stimuli that enhance an organism’s response to recurrent exposure to the same stimuli (D'Urso and Brickner, 2014). In this chapter, we assess the potential involvement of “transcriptional memory” in AKI-to-CKD transition by reviewing the most recent studies on the regulatory mechanism of gene transcription that serve as the basis of transcriptional memory. The eukaryotic synthesis of mRNAs and noncoding RNAs by RNA polymerase II (Pol II) comprises the layers of transcriptional regulation as follows: chromatin opening, transcription initiation, and elongation (Cramer, 2019).

### 4.1 Chromatin accessibility

#### 4.1.1 Chromatin accessibility is regulated by distinct classes of promoters and transcription factors

The first event in gene transcription is the assembly of Pol II and the general transcription factors (GTFs), including TFIIB, TFIID, TFIIE, TFIIF, and TFIIH into a preinitiation complex (PIC) at the promoter domain (Muller and Tora, 2014; Sainsbury et al., 2015). For Pol II to initially access the promoter region, the chromatin state must be open (Cramer, 2019). Certain distinct classes of promoters regulate their target chromatin states; promoters containing CpG islands, often present in housekeeping genes, are generally unmethylated and destabilize nucleosomes to generate a transcriptionally permissive chromatin state (Deaton and Bird, 2011; Schubeler, 2015). Chromatin opening at promoter regions is regulated as well by TFs that bind near the promoter or to enhancers in a sequence-specific manner (Fong et al., 2012; Cramer, 2019). These events regulate promoter accessibility through the function of their binding partners such as histone modifiers and chromatin remodelers (Fong et al., 2012; Cramer, 2019). Further, most TFs only bind to physically accessible, nucleosome-depleted DNA; therefore, local accessibility states must be established to initially bind TFs (Klemm et al., 2019).

#### 4.1.2 TFs gain access to closed chromatin through several mechanisms

The mechanism underlying the ability of a TF to access closed chromatin is insufficiently understood despite evidence that suggest several models of chromatin accessibility remodeling for TF binding (Klemm et al., 2019). First, TFs replace nucleosomes by exploiting the short period of DNA accessibility during nucleosome turnover and subsequently provide a recruitment substrate for cofactors and other TFs to stabilize the accessible state (Klemm et al., 2019). Increasing the concentrations of TFs and active chromatin remodelers that modulate nucleosome turnover rates raises the possibility of such replacement and promotes accessibility remodeling (Bao et al., 2015). Further, in concert with active chromatin remodelers, TFs transiently bound to internucleosomal DNA initiate proximal nucleosome removal in cis (Vicent et al., 2011; Lone et al., 2013; Klemm et al., 2019). Moreover, TFs bound to accessible enhancers mediate distal chromatin remodeling in trans by recruiting chromatin remodelers and other TFs (Taberlay et al., 2011; Klemm et al., 2019). Finally, a distinct class of “pioneer” TFs can bind to nucleosomal DNA in closed chromatin and trigger remodeling to provide accessibility to other TFs, leading to the formation of activating regulatory sequences (Mayran and Drouin, 2018; Cramer, 2019; Zaret, 2020).

As reviewed above, promoter accessibility must be established to initiate transcription, and highly dynamic chromatin accessibility is regulated by the interactions among TFs, active chromatin remodelers, and histone modifiers (Klemm et al., 2019), which are required to achieve the epigenetic regulation of gene transcription.

### 4.2 Transcription initiation

#### 4.2.1 Transcription initiation begins with PIC assembly

**FIGURE 2:**
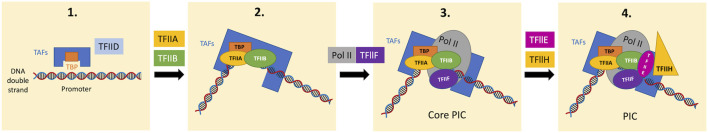
Stepwise PIC assembly from GTFs and Pol II on the promoter domain. TFIID contains TBP and several TBP-associated factors (TAFs). In the first step, the TBP subunit of TFIID recognizes the TATA box of the promoter domain in a sequence-specific manner (promoter recognition), bending the DNA by 90°. The TBP-DNA complex is stabilized by TFIIB and the auxiliary factor TFIIA. The Pol II-TFIIF complex then binds the TFIIB-TBP-DNA complex, forming the core PIC. Subsequently, TFIIE and TFIIH bind to the core PIC to form the complete PIC. In the presence of ATP, the XPB subunit of TFIIH, hydrolyses ATP to unwind DNA and propel it into the Pol II active center (DNA opening), which allows the initiation of RNA synthesis. TBP, TATA box-binding protein; TAFs, TBP-associated factors; PIC, preinitiation complex.

Returning to the promoter recognition in PIC assembly, the TATA box-binding protein (TBP) subunit of TFIID binds to the TATA box in promoters in a sequence-specific manner (Figure 2) (Sainsbury et al., 2015). Although most promoters lack canonical TATA boxes, proper promoter recognition may be achieved through other mechanisms such as sensing the flanking +1 nucleosome by TFIID and reading out features of DNA shape (Vermeulen et al., 2007; Sainsbury et al., 2015; Levens et al., 2016; Cramer, 2019).

The promoter-TBP complex then binds to TFIIB, which is required for initiation through the recruitment of the Pol II-TFIIF complex to the promoter with its N-terminal domain (“B-ribbon”) bound to Pol II and its C-terminal domain (“B-core”) bound to DNA and TBP (Kostrewa et al., 2009; Cramer, 2019). TFIIB also stimulates initial RNA synthesis through the contribution to mechanisms including DNA opening, the initiation of RNA synthesis, and stabilization of an early initiation complex (Sainsbury et al., 2013; Sainsbury et al., 2015; Cramer, 2019).

Thereafter, this core initiation complex assembles with TFIIE and TFIIH and forms a complete PIC (Figure 2) (Sainsbury et al., 2015). The TFIIH subunit, DNA translocase (xeroderma pigmentosum B; XPB), is required for subsequent DNA opening; XPB unwinds downstream DNA and propels it into the Pol II active center (Grunberg et al., 2012; Cramer, 2019). DNA opening leads to the formation of the open promoter complex containing the DNA template strand in the Pol II active site, which allows DNA-dependent RNA synthesis (Cramer, 2019).

#### 4.2.2 Mediator is required for Pol II to initiate transcription

**FIGURE 3:**
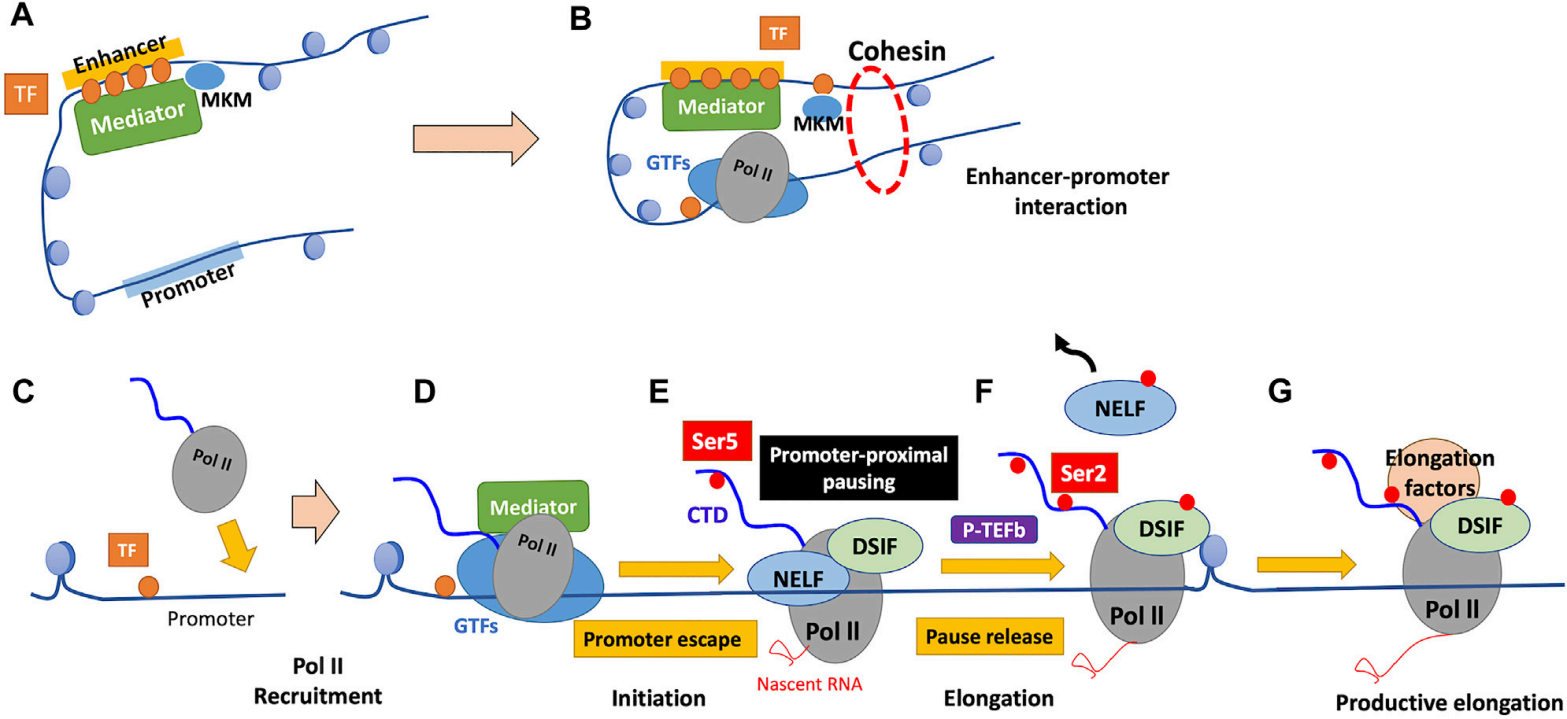
Mechanism of regulation of transcription by Pol II. **(A)** TFs bound to enhancers recruit chromatin remodelers and Mediator. Mediator comprises the core Mediator and dissociable Mediator kinase module (MKM). Binding of MKM to the core Mediator, which is mutually exclusive with the kinase module of TFIIH (TFIIK), inhibits PIC assembly and transcription initiation. **(B)** Dissociation of MKM from the core Mediator allows recruitment of TFIIK to the PIC, enabling subsequent transcription initiation. The enhancer-bound Mediator is held in proximity to the promoter by a cohesin-mediated loop structure, which contributes to the enhancer-promoter interaction. **(C)** TFs bind near the promoter in a sequence-specific manner, which ensures promoter accessibility through the function of their binding partners. **(D)** GTFs and Pol II are recruited to form the PIC, and the CTD of Pol II remains unphosphorylated. **(E)** The phosphorylation of Ser5 of the Pol II CTD by TFIIK facilitates promoter escape. Subsequent to initiation, Pol II typically pauses 20–80 nucleotides after the TSS, and Pol II is stabilized there by the pausing factors (DSIF and NELF). **(F)** Pause release requires the phosphorylation of DSIF and NELF by P-TEFb, accompanied by Ser2 phosphorylation of the Pol II CTD. Following pause release, Pol II soon encounters the first nucleosome that serves as a structural barrier against elongation. **(G)** Various elongation factors and histone modifiers recruited to Pol II alleviate nucleosome barriers, enabling subsequent productive elongation. GTFs, general transcription factors; TF, transcription factor; CTD, C-terminal domain; NELF, negative elongation factor; DSIF, DRB sensitivity-inducing factor; P-TEFb, positive transcription elongation factor b.

The multi-subunit coactivator complex Mediator regulates the initiation phase of transcription in various aspects (Kornberg, 2005; Venters and Pugh, 2009). Mediator comprises head, middle, tail, and kinase modules; the core Mediator, comprising the head and middle modules, binds Pol II and GTFs, while the tail module contacts sequence-specific activating TFs (Lacombe et al., 2013; Cramer, 2019; El Khattabi et al., 2019). Mediator acts as a functional bridge between enhancers and promoters, which is essential for transmission of functional information from enhancer-bound TFs to the basal Pol II machinery at promoters (Figures 3A,B) (Lacombe et al., 2013; El Khattabi et al., 2019). When the transcribed RNA reaches a certain length, Mediator activates the TFIIH cyclin-dependent kinase subunit CDK7, which phosphorylates the serine-5 (Ser5) residues of tandem heptapeptide repeats of the C-terminal domain (CTD) of Pol II (Jeronimo and Robert, 2017; Cramer, 2019). Ser5 phosphorylation of the CTD of Pol II facilitates a step called “promoter escape” by disrupting Mediator-Pol II interactions, which is required for transcription to proceed (Figure 3D) (Jeronimo and Robert, 2017).

### 4.3 Transcription elongation

#### 4.3.1 Pol II transcription is prone to arrest during early elongation

After promoter escape, Pol II forms the elongation complex that processively extends the nascent RNA chain (Cramer, 2019). During early elongation, Pol II is prone to arrest in a “backtracked” position, in which the catalytic site of Pol II is displaced upstream of the 3′ terminus of nascent RNA (Kwak and Lis, 2013). The elongation factor TFIIS, which rescues arrest, binds to arrested Pol II and stimulates Pol II to cleave protruded RNA to reconstitute a new 3′ terminus with the catalytic site for the restart of elongation (Cheung and Cramer, 2011; Kwak and Lis, 2013).

#### 4.3.2 Pol II must pass through “promoter-proximal pausing” to mediate productive elongation

In metazoan cells, Pol II typically pauses 20–80 nucleotides after the TSS, which is referred to as “promoter-proximal pausing” (Cramer, 2019; Luyties and Taatjes, 2022). Such pausing involves adapting the nonproductive conformation of the DNA-RNA hybrid, which is stabilized by the pausing factors DRB (5,6-dichloro-1-β-d-ribofuranosyl-benzimidazole) sensitivity-inducing factor (DSIF), a heterodimer comprising the elongation factor Spt5 and its binding partner Spt4, and negative elongation factor (NELF) (Figure 3E) (Core and Adelman, 2019). Release of paused Pol II to achieve productive elongation requires the phosphorylation of these two pausing factors and release of NELF by the kinase CDK9, a subunit of the positive transcription elongation factor b (P-TEFb), which is required for productive elongation (Kwak and Lis, 2013; Core and Adelman, 2019; Cramer, 2019). P-TEFb also phosphorylates Ser2 of the Pol II CTD, a mark of active and productive elongation, which recruits positive elongation factors (Kwak and Lis, 2013; Core and Adelman, 2019). Ser2 phosphorylation by P-TEFb is efficient only in the presence of phosphorylated Ser7, which is deposited by CDK7 during the initiation phase, suggesting that it occurs only if transcription is successfully initiated with Pol II CTD “primed” for P-TEFb recognition (Czudnochowski et al., 2012; Core and Adelman, 2019). Whereas 7SK snRNP (small nuclear ribonucleoprotein) inhibits unbound P-TEFb, the latter is released and recruited to paused Pol II on activation (Kwak and Lis, 2013). P-TEFb is then inhibited again by 7SK; however, in highly transcriptionally active genes, it remains bound to Pol II in super elongation complexes (SECs) (Luo et al., 2012; Kwak and Lis, 2013). P-TEFb is recruited to paused Pol II through TFs, Mediator, and the BET protein BRD4: 1. TFs on promoters directly interact with P-TEFb; 2. Mediator serves as well as a binding platform for SEC containing P-TEFb; and 3. BRD4 binding to acetylated histone tail recruits P-TEFb through its extended CTD (Kwak and Lis, 2013; Core and Adelman, 2019). BRD4 also releases P-TEFb from 7SK and stimulates P-TEFb kinase activity; this allosteric activation of P-TEFb is attracting attention as an important contribution of BRD4 to the transcriptional machinery (Schroder et al., 2012; Itzen et al., 2014; Core and Adelman, 2019).

#### 4.3.3 Elongation factors mediate the passage of Pol II through the gene body

Following its escape from promoter-proximal pausing, Pol II moves downstream to the gene body and soon encounters nucleosomes as structural barriers against elongation (Figure 3F) (Core and Adelman, 2019). Elongation factors, including polymerase-associated factor 1 (PAF1), facilitates chromatin transcription (FACT), and SPT6, alleviate such nucleosome barriers (Figure 3G) (Kwak and Lis, 2013; Core and Adelman, 2019). PAF1 serves as a platform for recruiting elongation factors and histone modifiers (Kwak and Lis, 2013; Vos et al., 2018). The histone chaperone activity of FACT and SPT6 facilitate disassembly and reassembly of nucleosomes during passage of Pol II through the gene body (Core and Adelman, 2019).

### 4.4 Factors that contribute to the efficiency of gene transcription

#### 4.4.1 Promoter-proximal pausing allows fine-tuning of gene expression

Promoter-proximal pausing reviewed above is a widespread transcriptional regulatory mechanism employed by higher eukaryotes (Adelman and Lis, 2012; Liu et al., 2020). Global run-on sequencing (GRO-seq) is a method that shows high-resolution mapping of Pol II engaging transcription, and GRO-seq in human primary lung fibroblasts reveals that approximately 30% of all genes exhibit paused Pol II (Core et al., 2008; Adelman and Lis, 2012). Studies in other species, including mouse embryonic stem cells and *Drosophila* cells, suggests that the proportion of genes displaying Pol II pausing is roughly conserved across species (Larschan et al., 2011; Min et al., 2011; Adelman and Lis, 2012). Further, only <1% of genes with paused Pol II are transcriptionally inactive, suggesting that promoter-proximal pausing is not a mechanism for silencing gene expression but that for tuning gene expression as the rate-limiting step for subsequent productive elongation (Core et al., 2008; Adelman and Lis, 2012).

Pausing of Pol II allows fine-tuning of gene expression level through separate regulation of initiation and elongation (Core and Adelman, 2019). Pause release is leaky, and therefore a signaling pathway that exclusively stimulates initiation leads to low levels of pause release as basal expression (Core and Adelman, 2019). Stimulation of pause release alone does not lead to gene expression; high levels of initiation and pause release in the presence of both signals confer robust transcriptional activation (Duarte et al., 2016; Core and Adelman, 2019). This pausing is particularly highly enriched for genes that encode components of stimulus-responsive pathways, and pausing may contribute to priming rapid gene reactivation in response to the second stimulus (Gilchrist et al., 2012). Moreover, paused Pol II competes with nucleosomes for occupancy of promoters, contributing to subsequent activation by securing local chromatin accessibility (Gilchrist et al., 2010).

#### 4.4.2 Enhancer-promoter interactions mediate pause release

As described above in Section 4.3.2, P-TEFb recruitment is essential for pause release and resultant fine-tuning of gene expression (Core and Adelman, 2019; Liu et al., 2020). Further, other factors contribute to the regulation of pause release. Enhancer-promoter interactions mediated by cohesin and Mediator contribute to pause release in several ways (Chen et al., 2018). Cohesin, which forms a ring-like structure that holds an enhancer and a promoter together, is suggested to facilitate pause release (Figure 3B) (Schaaf et al., 2013; Izumi et al., 2015; Chen et al., 2018). Further, Mediator inhibits Gdown1 that stabilizes Pol II in a paused state (Cheng et al., 2012; Chen et al., 2018). Other evidence indicates that active enhancers, rich in acetylated H3K27 (H3K27ac), directly contribute to pause release; enhancer RNAs (eRNAs), a class of lncRNAs produced by active enhancers, may displace NELF from promoters to facilitate pause release (Schaukowitch et al., 2014; Chen et al., 2018). Increased levels of histone acetylation also contribute to the recruitment of BRD4 and SEC complexed with P-TEFb (Chen et al., 2018).

#### 4.4.3 Other factors that regulate pause release, including epigenetic modifications

Recent studies identify other regulators of pause release, including factors involved in DNA replication and the DNA damage response. For example, topoisomerase and poly (ADP-ribose) polymerases (PARPs) contribute to this regulatory step of elongation (Chen et al., 2018). The role of epigenetic modifications in pause release is exemplified by H3K9ac, a histone mark for active gene promoters, which facilitates pause release by directly recruiting SEC to chromatin; while H3K4me3 promotes initiation, H3K9ac mediates a switch from initiation to elongation (Gates et al., 2017). Further, the histone deacetylase SIRT6, as a pausing-dedicated histone deacetylase, interacts with Pol II and inhibits pause release by decreasing the levels of H3K9ac and H3K56ac (Etchegaray et al., 2019).

The persistent alterations of factors involved in these regulatory processes after initial stimulation may contribute to remodeling subsequent gene expression as epigenetic memory. For example, DNA methylation in the promoter region suppresses gene expression by either reducing chromatin accessibility for TFs or reader proteins that are involved in recruiting repressor complexes (Guo et al., 2019). Histone acetylation relaxes the chromatin structure by neutralizing the positive charge of lysine residues in histone tails and acetylated lysine residues serve as docking sites for transcriptional activators (Tanemoto and Mimura, 2022). H3K27ac is considered a mark for an active enhancer. Histone methylation also provides docking sites for transcriptional activators or repressors depending on the site and state of methylation. For example, histone methylation on H3K4, H3K36, and H3K79 is a mark for active transcription, whereas that on H3K9, H3K27, and H4K20 is associated with repressed transcription (Black et al., 2012).

## 5 Transcriptional memory: Possible contributor to late-onset diseases

### 5.1 What is transcriptional memory?

Transcriptional memory is a highly conserved molecular mechanism that influences the rate or output of gene expression following a stimulus and is enhanced by previous exposure to the same stimulus to optimize gene expression patterns in a rapidly changing environment (D'Urso and Brickner, 2017; Kim et al., 2019). This primed state of transcription is retained after the initial signal subsides (Siwek et al., 2020).

Transcriptional memory requires changes in factors that control the degree of gene expression. Such factors are induced and conserved following the initial stimulus. In principle, the “memory” is retained in factors described above, which are involved in each step of transcriptional regulation. These factors, including TFs and those involved in chromatin opening, recruitment, initiation, elongation of Pol II, and enhancer-promoter interactions.

The inducible gene encoding inositol-1-phosphate synthase (INO1) in budding yeast serve as models for transcriptional memory; moreover, emerging evidence suggests the contribution of transcriptional memory to immune responses of human cells (D'Urso and Brickner, 2014; Kim et al., 2019). Here we consider established examples of transcriptional memory and provide an update regarding the underlying molecular mechanisms.

### 5.2 Established transcriptional memory in yeasts: INO1 memory

In budding yeast, INO1 transcriptional memory induced by previous inositol starvation primes the rapid reactivation of *INO1* upon exposure to the second stimulus (D'Urso and Brickner, 2017). INO1 is required for the conversion of glucose to myoinositol in phospholipid biosynthesis (Graves and Henry, 2000; Sump et al., 2022). The detailed molecular mechanism of INO1 memory is under investigation, and the current model of INO1 memory is summarized in a recent study (Sump et al., 2022). Briefly, INO1 memory requires a TF-dependent interaction with the nuclear pore complex (NPC), alterations in chromatin structure, and recruitment of “poised” Pol II to the promoter.

On initial activation, *INO1* repositions from the nucleoplasm to the nuclear periphery and interacts with the NPC (Sumner et al., 2021; Sump et al., 2022). During subsequent repression, *INO1* continues to interact with NPC, which requires the transcription factor Sfl1, the memory recruitment sequences (MRS) in the promoter, to which Sfl1 specifically binds, and the nuclear pore protein Nup100 (Light and Brickner, 2013; D'Urso et al., 2016; Sump et al., 2022).

Recently repressed *INO1* acquires the essential histone mark H3K4me2 within its promoter domain (D'Urso and Brickner, 2017). Similarly, H2A.Z is incorporated upstream of the TSS during INO1 memory (Brickner et al., 2007; Sump et al., 2022). Active gene promoters generally possess high levels of histone acetylation, H3K4me3, and H3K4me2; H3K4 methylation is catalyzed by the SET1/COMPASS complex, which is the sole methyltransferase of H3K4 in yeast (Shilatifard, 2012; D'Urso and Brickner, 2017; Chen et al., 2018). Upon repression, TF binding remodels the COMPASS complex to remove the Spp1 subunit, which is required for trimethylation of H3K4, resulting in formation of the histone mark H3K4me2 at the promoter without H3K4me3, which is mitotically inherited for as many as 4 cell divisions (D'Urso et al., 2016; D'Urso and Brickner, 2017; Sump et al., 2022). SET3C, a histone deacetylase complex that recognizes H3K4me2, is essential for maintaining this histone mark; SET3C physically interacts with COMPASS lacking Spp1 (Spp1^-^ COMPASS), which may involve H3K4me2 inheritance during DNA replication (D'Urso et al., 2016; Sump et al., 2022). Moreover, Nup100 may be required for memory-associated H3K4me2 in INO1 memory through the recruitment of Spp1^-^ COMPASS, as well as the interaction with *INO1* (Pascual-Garcia et al., 2014; Sump et al., 2022).

Another feature of INO1 memory is persistent binding of a “poised” form of Pol II at the promoter, which bypasses Pol II recruitment and leads to prompt reactivation on subsequent reactivation (D'Urso and Brickner, 2017; Sump et al., 2022). Among “poised” Pol II, the generic term for Pol II located near the TSS, INO1 memory is associated with recruited Pol II with its unphosphorylated CTD that has not initiated transcription (Figure 3D) (Adelman and Lis, 2012; D'Urso and Brickner, 2017). This poised Pol II PIC is characterized by the recruitment of CDK8 (Ssn3 in yeast)-containing Mediator and lack of CDK7 (Kin28 in yeast)-containing TFIIK, the TFIIH kinase module (D'Urso and Brickner, 2017; Sump et al., 2022). CDK8 is a conserved subunit of the dissociable Mediator kinase module that negatively and positively regulates transcription (Donner et al., 2010; Osman et al., 2021; Luyties and Taatjes, 2022). Upon initiation, the Mediator kinase module binding to core Mediator is mutually exclusive with TFIIK; thus, Mediator bound to the kinase module (CDK8-Mediator) sterically blocks recruitment of TFIIK to the PIC to inhibit subsequent CDK7-dependent phosphorylation of Pol II CTD–Ser5 and initiation (Tsai et al., 2017; Rengachari et al., 2021; Luyties and Taatjes, 2022). In contrast, CDK8 phosphorylation may promote dissociation of Mediator kinase module from core Mediator, which then promotes PIC assembly and transcription initiation (Osman et al., 2021; Luyties and Taatjes, 2022). The dissociated kinase module remaining associated with TFs at enhancers may regulate pause release and elongation through phosphorylation of P-TEFb/SEC (Luyties and Taatjes, 2022). During the formation of INO1 memory, a specific TF remodels Mediator to recruit CDK8, which inhibits recruitment of CDK7-containing TFIIK, resulting in recruitment of unphosphorylated poised Pol II at the promoter (D'Urso and Brickner, 2017; Sump et al., 2022).

The critical features of INO1 memory, including interaction with the nuclear pore protein Nup100, histone mark H3K4me2, and binding of poised Pol II at the promoter, are evolutionarily conserved and widespread (D'Urso and Brickner, 2017; Sood et al., 2017; Sump et al., 2022). Evidence indicates that in yeast GAL memory (galactose-induced transcriptional memory in *GAL* genes: *GAL1*, *GAL2*, *GAL7*, and *GAL10*), along with other mechanisms, epigenetically inheritable changes such as the incorporation of H2A.Z, H3K4me2, and poised Pol II at the promoter contribute to primed reactivation (Sood et al., 2017; Cerulus et al., 2018; Bheda et al., 2020). Further, the primed reactivation of oxidative stress-induced genes in yeast previously exposed to high salt concentration requires interaction with nuclear pore protein (Nup42 instead of Nup100) (Guan et al., 2012; D'Urso et al., 2016).

### 5.3 Transcriptional memory is evolutionarily conserved and identified in mammals as well

#### 5.3.1 “Conservation” of transcriptional memory at different regulatory steps of transcription

Hundreds of IFNγ-inducible genes exhibit enhanced gene expression in the human HeLa cell line previously exposed to IFNγ (D'Urso and Brickner, 2017). The interaction of genes with the nuclear pore protein Nup98 (homologous to yeast Nup100), H3K4me2, and retention of poised Pol II on promoters represent the molecular features of IFNγ memory (Figure 3D) (Gialitakis et al., 2010; Light et al., 2013; D'Urso and Brickner, 2017). IFNβ-stimulated mouse embryonic fibroblasts (MEFs) and IFNγ-stimulated bone marrow macrophages exhibit a similar memory response (Kamada et al., 2018). However, retention of poised Pol II at promoters likely does not contribute to the memory in this case, which is attributed to accelerated recruitment of Pol II and the TF downstream of IFN stimulation (phosphorylated STAT 1; pSTAT1) (Kamada et al., 2018). These events are accompanied by the replacement of histone H3.3 and the H3K36me3 histone mark, which persistent as chromatin features of genes with IFNγ-induced memory (Kamada et al., 2018). Further, IFNγ memory in human cells reveals important properties of this memory (Siwek et al., 2020). First, single-cell RNA-seq indicates that the dominant mechanism of enhanced overall expression upon second exposure increases in the population of activated cells, rather than the transcriptional output per cell (Siwek et al., 2020). Second, in cells with the gene encoding guanylate binding protein 5 (GBP5) primed, which are stimulated by IFNγ, chromatin features such as H3K4me2 and H3.3 are relatively short-lived, suggesting that other factors maintain IFNγ memory associated with the genes examined (Siwek et al., 2020). Finally, strongly primed genes tend to be localized in genomic clusters restricted by cohesin, and the cohesin-controlled local chromatin structure may serve as an important contributor to IFNγ memory (Figure 3B) (Siwek et al., 2020).

Evidence indicates that IFNγ memory underlies the broader physiological phenomenon of “trained immunity,” in which an innate immune response triggered by previous exposure to a pathogen is retained as an inactive poised state, leading to an enhanced reaction in response to a second stimulus (Netea et al., 2020; Siwek et al., 2020). For example, analysis of immunity in the imiquimod (IMQ) model of skin inflammation using murine epidermal stem cells shows that chromatin accessibility gained during the inflammation phase is retained following resolution, which is maintained by the function of the TF remaining bound to this memory domain (Larsen et al., 2021). This study provides mechanistic insights into how primed genes are reactivated following exposure to diverse secondary stimuli (Larsen et al., 2021). During initial inflammation, a stimulus-specific TF such as STAT3 specifies the memory domain and recruits the FOS-JUN universal stress-responsive TF, which is required for establishing accessible chromatin by recruiting the SWI/SNF chromatin remodeling complex, which in turn establishes open chromatin that remains occupied by JUN in the absence of transcription (Vierbuchen et al., 2017; Larsen et al., 2021). Therefore, FOS is rapidly re-recruited to these scaffolds in the absence of the initial stimulus-specific TF, achieving robust gene expression in response to a diverse secondary stimulus (Larsen et al., 2021). This study suggests that transcriptional memory may be conserved at the level of TFs that regulate chromatin accessibility (Figure 3C).

Moreover, in heat-shocked MEFs transcriptional memory is induced by releasing paused Pol II that is increased by preceding stimuli upon reactivation (Vihervaara et al., 2021). These findings indicates that promoter-proximal paused Pol II may serve as a “holder” of transcriptional memory (Figure 3E). Further, retained changes in chromatin accessibility may be involve in transcriptional memory induced by glucocorticoid (GC) signaling. Several studies indicate that glucocorticoid receptor (GR)-induced chromatin alterations persist after the withdrawal of GCs (Zaret and Yamamoto, 1984; Stavreva et al., 2015; Jubb et al., 2017), which may regulate gene expression possibly by altering enhancer activity (Stavreva et al., 2015). In GC signaling memory, chromatin accessibility may serve as a “holder” of transcriptional memory (Figure 3C).

#### 5.3.2 Transcriptional memory may be associated with late-onset diseases through the cumulative effects of repeated stimuli

The examples of transcriptional memory with their assumed “holders” of the memory described above, indicates that such mechanisms serve to adapt a cell to more effectively respond to a secondary stimulus such as inflammation. However, this may represent a maladaptive process under certain conditions (Larsen et al., 2021). For example, epigenetic alterations during early life may gradually manifest as late-onset diseases such as chronic inflammation caused by the effects of repeated stimuli (Larsen et al., 2021). Similar to the reaction to inflammation, transcriptional memory may contribute to the pathophysiology of AKI-to-CKD transition. Transcriptional memory may mediate an adaptive response during kidney repair upon recurrent AKI, through increasing profibrotic gene expression as described in Section 2.4.1. However, these events may be associated with the progression of tubulointerstitial fibrosis through cumulative effects that are characteristic of chronic inflammatory diseases. Indeed, recurrent episodes of AKI confer a cumulative risk for developing CKD (Thakar et al., 2011).

#### 5.3.3 Tubular epithelial cells may utilize the mechanism of transcriptional memory

Bansal et al. recently conducted multi-omics analyses of gene expression, DNA methylation, and chromatin accessibility of kidney proximal tubular epithelial cells (PTECs) collected from subjects with T2DM (T2D-PTECs) and without T2DM (N-PTECs) (Bansal et al., 2020). They demonstrated epigenetic memory that continuously activates gene expression and contributes to “metabolic memory” in DKD, which is stored in T2D-PTECs. Compared with N-PTECs, T2D-PTECs exhibit persistent changes in gene expression and epigenetic profiles after long-term *in vitro* culture under nondiabetic conditions (Bansal et al., 2020). Notably, comparing the responses to TGFβ1 exposure as a DKD-related secondary stimulus after *in vitro* culture between T2D-PTECs and N-PTECs, T2D-PTECs exhibit an enhanced expression of specific TGFβ1-regulated genes, suggesting epigenetic memory that induces primed reactivation (Bansal et al., 2020), possibly mediated by a mechanism similar to transcriptional memory reviewed in this chapter. Further studies must identify the underlying mechanisms of such persistent epigenetic changes. Thus, persistent binding of TFs and Pol II at the promoter as well as enhancer-promoter interactions may represent possible contributors.

Moreover, transcriptional memory may be induced in tubular epithelial cells with “endotoxin preconditioning”. The mechanisms of endotoxin preconditioning have been extensively studied in immune cells, such as macrophages. Toll-like receptors (TLRs) exposed to bacterial endotoxin, such as LPS, induce the transient silencing of a subset of genes, including those encoding proinflammatory mediators, and exhibit a transient immunosuppressive state upon recurrent stimulation with LPS (“LPS tolerance”). This is one of the most important mechanisms that protects the host from dysregulated inflammation (Foster et al., 2007; López-Collazo and del Fresno, 2013; Boonmee et al., 2021). Foster et al. showed that several hundred genes, including those encoding antimicrobial effectors, induced by the initial exposure to LPS stimulation, exhibit enhanced expression upon subsequent LPS stimulation. This suggests that preconditioned macrophages utilize the mechanism of transcriptional memory (Foster et al., 2007). In sepsis models, Hato et al. demonstrated that endotoxin preconditioning not only increases survival but also preserves kidney function and reduces tubular injury (Hato et al., 2018). This protective preconditioning is achieved by the contributions of a set of macrophages and S1 segments of the proximal tubules. The clustering of active M2 macrophages around S1, promoted by a local chemokine gradient that is generated via robust expression by preconditioned S1 PTECs, mediates the renoprotective effect (Hato et al., 2015; Hato et al., 2018). Indeed, a set of chemokines exhibits enhanced expression upon LPS stimulation in PTECs with endotoxin preconditioning compared with those without preconditioning, which may be achieved by the mechanism of transcriptional memory as well as preconditioned macrophages (Foster et al., 2007; Hato et al., 2018).

These studies suggest that renal tubular epithelial cells may also utilize the mechanism of transcriptional memory. It is quite possible that transcriptional memory is also involved in the expression of genes that promote inflammation and fibrosis in the pathogenesis of AKI-to-CKD transition as well.

## 6 Hypothetical epigenetic memory contributing to AKI-to-CKD transition

Numerous studies show that the molecules that confer epigenetic modifications involved in the pathophysiology of AKI-to-CKD transition show promise as future therapeutic targets (Fontecha-Barriuso et al., 2018; Guo et al., 2019; Li and Li, 2021; Tanemoto and Mimura, 2022). However, insufficient data are available on the retained epigenetic modifications (“epigenetic memory”) of AKI that contribute to the progression to CKD. It is difficult to distinguish whether epigenetic modifications serve as a causative driver or represent a consequence of transcriptional regulation (Zager and Johnson, 2010). However, evidence supports that AKI induces nucleosome remodeling that affects subsequent gene expression (Reddy and Natarajan, 2015; Zhuang, 2018). Moreover, a hypothesis is plausible that epigenetic alterations that cause proregenerative phenotypic changes during injury repair maladaptively remain in response to severe or repetitive insults, which result in progressive tubulointerstitial fibrosis. Therefore, the potential role of epigenetic memory in the pathophysiology of AKI-to-CKD transition requires further study.

As described in Section 3.2 and 3.3, several studies show that epigenetic alterations, including histone modifications and DNA methylation, occur throughout the observational periods that alter the regulation of gene expression during AKI-to-CKD transition (Zager et al., 2011; Zhao et al., 2017; Heylen et al., 2018; Sanchez-Navarro et al., 2021). Further, dynamic phenotypic changes of dedifferentiated tubular epithelial cells, which are required for the initiation of kidney repair, are mediated by epigenetic changes and resultant enhancer and SE dynamics (Wilflingseder et al., 2020). Generally, enhancers primarily encode cell-type specificity of gene expression (Larsson et al., 2019). Similarly, the recently identified subset of cells called “failed repair” proximal tubular cells, may be associated with epigenetic alterations, particularly those associated with enhancer regulation, which may represent “epigenetic memory” that maladaptively persists from the early dedifferentiated cell after injury. The age-related increase in specific cell types, such as “failed repair” proximal tubular cells and “*VCAM1* ^*+*^ proximal tubular cells” identified in healthy human kidneys (Muto et al., 2021; Little and Humphreys, 2022), suggests that epigenetic modifications influence their characteristic gene expression profiles also suggests epigenetic involvement in their characteristic gene expression patterns. It is therefore possible that epigenetic memory that induces persistent changes in their modes of regulating gene expression contributes to the transition from AKI to CKD.

Moreover, successfully repaired tubular epithelial cells without a persistent proinflammatory or profibrotic phenotypic change may possess underlying epigenetic memory that primes prompt and enhanced reactivation upon the second injury, e.g. transcriptional memory. Transcriptional memory is evolutionarily conserved (D'Urso and Brickner, 2014), and the underlying molecular mechanisms are being identified in human cells (e.g., IFNγ memory as reviewed in the previous chapters) as well as in model organisms such as yeasts. Moreover, human proximal tubular epithelial cells may exhibit transcriptional memory. For example, PTECs collected from T2DM patients exhibit enhanced TGFβ1-regulated gene expression upon a DKD-related secondary stimulus (TGFβ1) after *in vitro* culture under nondiabetic conditions (Bansal et al., 2020). Upon LPS stimulation, preconditioned S1 PTECs exhibits enhanced expression of a set of chemokines (Hato et al., 2018). Similarly, it is reasonable to hypothesize that fibrogenic factors required for wound repair are induced more rapidly and more robustly to achieve more effective repair upon the second AKI. Although a single stimulus may induce the acquisition of such epigenetic memory in only a small subset of tubular cells, recurrent episodes of AKI may increase the population of tubular cells primed for reactivation through cumulative effects, enhancing overall expression of profibrotic genes upon the next AKI episode. The accumulation of enhanced profibrotic responses to recurrent and even mild AKI and hypoxic stress may promote the progression of tubulointerstitial fibrosis (Mimura, 2016).

**FIGURE 4:**
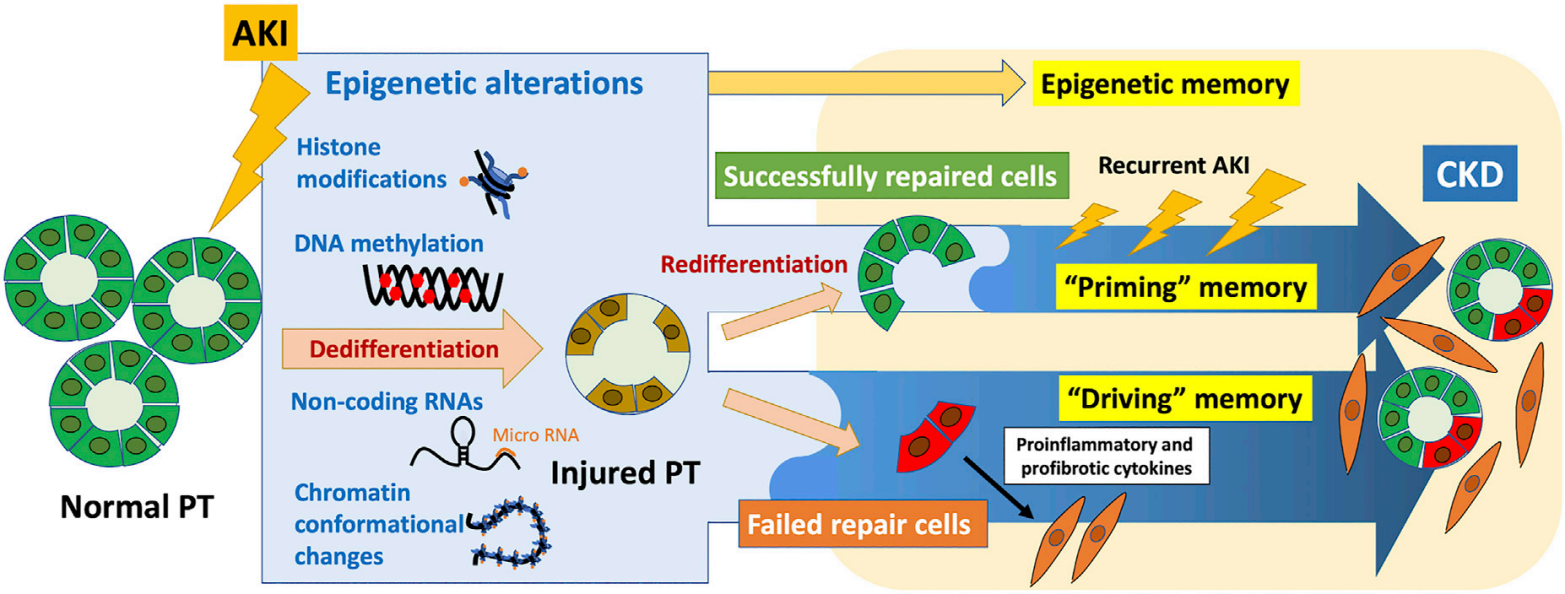
Contribution of epigenetic memory to AKI-to-CKD transition. Epigenetic alterations induced by AKI, including histone modifications, DNA methylation, noncoding RNAs, and chromatin conformational changes, are involved in cellular adaptive response to promote wound repair during the early phase after AKI. Some of these epigenetic alterations are possibly stored in tubular epithelial cells as epigenetic memory, which may contribute to the progression of tubulointerstitial fibrosis. “Priming” memory that enhances future transcriptional responses to restimulation may be stored in seemingly successfully repaired cells without persistent changes in gene expression. This type of epigenetic memory promotes tubulointerstitial fibrosis through cumulative effects after recurrent AKI or hypoxic episodes. “Driving” memory with persistent changes in associated regulated gene expression may be involved in forming specific cell clusters with proinflammatory and profibrotic phenotype, including “failed repair” proximal tubular cells. PT, proximal tubule.

In summary, we propose that epigenetic memory contributes to the pathophysiology of AKI-to-CKD transition through the events as follows (Figure 4): “driving” memory that persistently alters regulated gene expression and “priming” memory that enhances future transcriptional response to restimulation without persistent changes in gene expression. The former “driving” memory may contribute to the progression of tubulointerstitial fibrosis by continuously activating the transcription of fibrogenic genes or inhibiting that of renoprotective genes. As such “driving” memory may contribute to the maladaptive repair process of tubular epithelial cells after AKI through the formation of specific cellular clusters such as those recently identified as “failed repair” proximal tubular cells. The latter “priming” memory mediated by the same mechanism as evolutionarily conserved transcriptional memory may operate in apparently successfully repaired tubular epithelial cells in the absence of persistent changes in the level of proinflammatory or profibrotic gene expression. Such epigenetic memory may also promote tubulointerstitial fibrosis through its cumulative effects during recurrent AKI or hypoxic episodes. Both memories may contribute to AKI-to-CKD transition and therefore have great potential as novel therapeutic targets.

## 7 Discussion

Epigenetic memory acquired in response to environmental stimuli is increasingly recognized as a contributor to diverse late-onset diseases. In AKI-to-CKD transition, depending on the severity of AKI, a subset of AKI-induced epigenetic alterations is retained in cells, including tubular epithelial cells, which may be crucial drivers mediating subsequent progression of tubulointerstitial fibrosis.

Recent findings regarding the molecular mechanism of epigenetic memory and accumulating evidence of epigenetic involvement in the pathophysiology of AKI-to-CKD transition lead us to conclude that two types of epigenetic memory contribute to AKI-to-CKD transition, we designate here as “driving” memory and “priming” memory.

As a regulator of enhancer and SE dynamics, epigenetic alterations induce dynamic phenotypic changes in tubular epithelial cells during the early phase of kidney repair after AKI. As a maladaptive remnant of such epigenetic alterations, “driving” memory may persistently activate or inhibit gene expression, which promotes tubulointerstitial fibrosis during the chronic phase. The formation of specific cell clusters with phenotypic alterations, including recently identified “failed repair” proximal tubular cells, may be mediated by this “driving” memory.

Moreover, a subset of successfully redifferentiated tubular cells after injury that acquire epigenetic “priming” memory may exhibit enhanced expression of profibrotic genes required for wound repair upon the second AKI. This “priming” memory is retained within each regulatory step of Pol II transcription as follows: Pol II recruitment (regulated by TF binding, chromatin accessibility, and enhancer-promoter proximity), transcription initiation (regulated by factors mediating promoter escape), and elongation (regulated by factors mediating pause release). Alterations of these regulating factors caused by AKI that persist as “priming” memory may thereafter induce excessive responses to even a mild AKI, and the accumulation of such responses may lead to the progression of tubulointerstitial fibrosis.

Therapies targeting epigenetic mechanisms that underlie AKI-to-CKD transition show promise because of their reversibility and accumulating favorable results in preclinical studies. Along with recent technical advances in epigenetic editing, identifying the detailed mechanisms of epigenetic memory mediating the transition from AKI to CKD will help resolve the low specificity of potential therapeutics and their wide range of off-target effects, which pose major clinical challenges. Importantly, temporal specificity should be considered along with locus specificity for optimizing the epigenetic intervention of AKI-to-CKD transition. Emerging evidence suggests that fibrosis plays a proregenerative role in the early phase of kidney repair, and antifibrotic therapy may exert opposing effects on disease progression, depending on the timing of treatment. Therefore, the most suitable timing to maximize therapeutic effects by targeting maladaptively maintained epigenetic memory must be determined.

In conclusion, epigenetic memory that remains maladaptive after the completion of the tubular repair process shows great potential as a novel therapeutic target of AKI-to-CKD transition. Recent advances in structural and genome-wide analyses will illuminate in greater detail the epigenetic landscape during the transition from AKI to CKD. Timely, target-specific epigenetic intervention established according to a detailed understanding of epigenetic memory will resolve the challenges of low-specificity and broad off-target effects, and present the possibility of applying epigenetics to clinical practice.
